# Spontaneous Pulp-Death

**Published:** 1866-12

**Authors:** Henry S. Chase

**Affiliations:** Iowa City


### SPONTANEOUS PULP-DEATH.

HENRY S. CHASE, D. D. S., IOWA CITY.

Mrs. P———, age 18, a brunette, soon after marriage, had pain of a throbbing character in both upper central incisors, which resulted in death of the pulps. Alveolar abscess formed from the roots of both teeth, which have continued to discharge about once a month ever since; (about seven years. These cases of spontaneous death are not common, and are usually produced by pathological conditions of the blood, or sudden changes of temperature.

These teeth were not decayed. I undertook their treatment about a month ago. One is thoroughly cured, and the other nearly so.

The treatment consisted in drilling a hole from the posterior surface, near the cutting edge in a direction which would correspond with the long axes of the pulp canals. Ten or fifteen drops of carbolic acid were forced through the canals of each, *without showing externally*; proving large cavities to exist in the alveoli. Alcohol was also forced in to dilute the carbolic acid, to moderate its escharotic action. Severe pain of a burning character was produced in both teeth at the first injection; and the day following the operation, there was extensive swelling of the gums, and soreness and looseness of the affecteed teeth. A discharge of pus and blood took place also through the openings I had made. In a week I made another injection, which caused *no pain*. Since then the purple and congested appearance of the gums is gone, and the projections of the fistulas have disappeared. The teeth are firm in their sockets. One was permanently plugged two weeks ago, and the other will probably be ready in a week.

In the treatment of alveolar abscess it seems desirable that the injection should appear externally; but in the cases which I have had in the last six months, I find that in eight cases in which the injection was made through the roots it did not show, yet every one was cured.

The abscess is sometimes so capacious that an amount which we would dare to inject, would not fill it and its canal. Yet I do not know that it would be dangerous to fill the cavity, even if it took a drachm or two, provided we leave open the hole in the the tooth for its evacuation, as the walls of the abscess contract. It is well known that carbolic acid, or creosote forms a chemical compound with albumen, which is not readily dissolved. Consequently, the action of these escharotics would be limited, in a measure, to the surface of the membranous walls of the abscess.

